# Survival after liver transplantation from hepatitis B-core positive donors at a quaternary care hospital in Brazil

**DOI:** 10.1016/j.bjid.2024.104384

**Published:** 2024-10-11

**Authors:** Fabiana Siroma, Edson Abdala, Stefanie Lima do Nascimento Castro, Wellington Andraus, Luiz Augusto Carneiro D´Álbuquerque, Alice Tung Wan Song

**Affiliations:** a Departamento de Doenças Infecciosas, Faculdade de Medicina, Universidade de São Paulo, São Paulo, SP, Brazil; b Departamento de Gastroenterologia, Faculdade de Medicina, Universidade de São Paulo, São Paulo, SP, Brazil

**Keywords:** Liver transplant, Anti-HBc-positive donor, Survival, Hepatitis B, Prophylaxis

## Abstract

**Background:**

Liver transplantation is the treatment for many end-stage liver diseases and hepatocellular carcinoma but shortage of available organs poses significant challenge. Many centers have used grafts from donors with positive anti-HBc serology but concerns about potential hepatitis B virus reactivation and *de novo* hepatitis B infection have raised questions about the safety of this approach. This study aimed to evaluate the survival of liver transplant recipients from anti-HBc-positive-donors and assess the risk of hepatitis B reactivation and *de novo* hepatitis B.

**Patients and methods:**

A retrospective single-center cohort study was conducted from 2002 to 2018, comparing who received grafts from anti-HBc-positive-donors to those from anti-HBc-negative-donors. The primary outcome was survival and description cases of hepatitis B reactivation/*de novo* hepatitis B.

**Results:**

We analyzed 1,111 liver transplants, in which 993 (89 %) received grafts from anti-HBc-negative-donors and 118 (11 %) from anti-HBc-positive-donors. Median age of recipients from anti-HBc-positive donors was 56 years and from anti-HBc-negative donors was of 53 years (*p* = 0.001). Male sex was predominant in both groups. Factors associated with death in multivariate analysis were retransplantation, early allograft dysfunction, high MELD, recipient over 60 years and female donor. The utilization of grafts from anti-HBc-positive-donors did not increase mortality. The majority of HBV reactivation and *de novo* hepatitis B occurred in anti-HBc positive recipients. The risk of hepatitis B reactivation/de novo hepatitis B was low and manageable.

**Conclusion:**

The study supports safety of liver grafts from anti-HBc-positive donors when employing antiviral prophylaxis. These findings contribute to expand donor options and improve patient outcomes

## Highlights

• Anti-HBc-positive liver graft donor was not a risk factor for lower survival.
• With prophylaxis, the occurrence of hepatitis B reactivation was low and manageable.
• Retransplant, early graft dysfunction and high MELD were linked to lower survival.
• Early allograft dysfunction rate was considered high among survivors and deaths.

## Introduction

Brazil ranks as the fourth leading country globally in absolute numbers of liver transplants, having performed 2044 liver transplant procedures in 2022. However, despite a notable rise in liver transplant numbers from 2009 to 2019, statistics from the Brazilian Association of Organ Transplants indicate that in 2019, fewer than 50 % of patients listed were transplanted. Notably, in 2022, the mortality rate for patients on the waiting list for liver transplantation was 26 %.^1^

Due to the shortage of organs and the increasing demand from patients on the waiting list, liver transplant centers have used grafts from anti-HBc donors to expand the pool of potential donors. Even though these donors do not show markers of active Hepatitis B Virus (HBV) infection, their livers may contain covalently closed circular DNA (cccDNA) and pre-genomic RNA in hepatocytes, which can be responsible for HBV reactivation and *de novo* hepatitis B.^2^ The risk of HBV transmission primarily depends on the serological status of the recipient and the adoption of prophylactic antiviral therapy.^3^ This therapy includes the use of specific Hepatitis B Immunoglobulin (HBIG), first-generation nucleos(t)ide analogs such as lamivudine and adefovir, and second-generation agents (entecavir and tenofovir).

In the absence of prophylaxis, the occurrence of *de novo* hepatitis B is low in recipients who are anti-HBc positive/anti-HBs positive and high in patients with no prior contact with HBV who are not vaccinated (anti-HBc and anti-HBs negative).^4^

Earlier studies conducted in the 1990s are controversial regarding the survival of patients and grafts from anti-HBc positive donors, before the routine adoption of antiviral prophylaxis and HBIG. However, most cases resulting in graft loss occurred for reasons other than *de novo* HBV or HBV reactivation. More recent studies, especially following the routine use of appropriate antiviral prophylaxis, have not demonstrated an increase in mortality in liver transplant recipients from anti-HBc positive donors.^5^, ^6^, ^7^ However, there is currently a lack of data in the literature regarding the survival of liver recipients in Brazil.

The present single center retrospective study compared the survival of liver transplant recipients from anti-HBc positive and anti-HBc negative donors, described cases of *de novo* hepatitis B and hepatitis B reactivation and mortality-related variables.

## Patients and methods

This was an observational, retrospective, single-center study of a cohort of patients aged 18 years or older who underwent liver transplantation at the Hospital das Clínicas, Universidade de São Paulo in Brazil between 2002 and 2018. Patients who died within the first 48 h after transplantation and those with insufficient medical record data were excluded.

### Data collection

The variables collected from recipients included gender, age, Body Mass Index (BMI), transplant date, MELD (Model for End-stage Liver Disease) score, diabetes mellitus, occurrence of hepatocellular carcinoma, retransplantation, portal vein thrombosis, transplant year, hepatitis B and C serology, date of the last follow-up, occurrence of Early Allograft Dysfunction (EAD), primary graft dysfunction, cold ischemia time and mortality. For patients with chronic hepatitis B, data on hepatitis B viral load before and after transplantation, HBIg prophylaxis, and antiviral use were assessed. Data from only one transplant per patient was considered. In the case of patients undergoing retransplantation, the study included data from the transplant with an anti-HBc positive donor or data from the last transplant in recipients without any anti-HBc positive donors.

The variables collected from the donors included age, gender, BMI, whether the donor was living, deceased, or had familial amyloidotic polyneuropathy, length of hospital stay before donation, Donor Risk Index (DRI), hepatitis B and C serology.

*De novo* hepatitis B refers to patients who were HBsAg-negative prior to transplantation and subsequently developed viremia and/or seroconversion to HBsAg positivity (regardless of their baseline anti-HBc status) or liver biopsy with positive immunohistochemistry for HBsAg or HBcAg. Hepatitis B reactivation was defined as new viremia in patients with previously undetectable viral load or an increase in viral load if previously viremic patients. Early Allograft Dysfunction (EAD) was defined using criteria described by Olthoff and colleagues,^9^ defined as the presence at least one of the following criteria: bilirubin ≥ 10 mg/dL on postoperative day 7; International Normalized Ratio (INR) ≥ 1.6 on postoperative day 7; alanine aminotransferase or aspartate aminotransferase > 2000 IU/L within the first postoperative 7 days. Primary graft dysfunction was defined as severe hepatic necrosis shortly after transplantation, characterized by rapid elevation of transaminases, coagulopathy, increased lactate levels, hemodynamic instability, and the need for urgent re-listing for liver transplantation.

### Protocols

All recipients of grafts from anti-HBc positive donors, regardless of previous serology, receive antiviral therapy according to current Clinical Protocols and Therapeutic Guidelines.^8^ Until August 2016, our institution performed liver transplantation from anti-HBc positive donors only for HBsAg or anti-HBc positive recipients. After 2016, we expanded the availability of anti-HBc positive donors to vaccinated recipients with anti-HBs > 10 UI/mL, regardless of anti-HBc status.

Prophylaxis to prevent *de novo* HBV infection was done with lamivudine (150 mg per day or dose adjusted according to renal function) until 2017 for all HBsAg-negative recipients of liver grafts from anti-HBc positive donors. Since 2017, lamivudine was progressively discontinued and replaced with tenofovir or entecavir. Chronic hepatitis B patients continued their antiviral treatment, except lamivudine and adefovir, which were similarly replaced with entecavir or tenofovir in 2017.

Chronic HBV patients, in addition to antivirals, also receive prophylaxis with HBIg (HBIg 800 IU intramuscularly during the intraoperative anhepatic phase, followed by 800 IU per day during the first 7 days post-transplant and 800 IU intramuscularly monthly for one year after the transplant).For all liver transplant patients, surgical prophylaxis includes ampicillin and cefotaxime for 24 h, or ampicillin and amikacin after 2015 in selected cases (colonization by multidrug resistant microorganism gram-negatives, MELD > 24, pre-transplant dialysis, use of broad-spectrum antimicrobials 30 days prior to transplantation). Antibiotic therapy is in therapeutic doses in case of previous active or suspected infection or confirmation of donor infection. Protocol immunosuppression includes intraoperative methylprednisolone or basiliximab in the presence of risk factors for post-transplant renal failure, and tacrolimus with or without mycophenolate sodium in addition to prednisone, tapered in 3‒6 months except in cases of autoimmune hepatitis or double liver-kidney transplant.

### Statistics and ethical statement

The statistical analyses were conducted using STATAversion 14.2 (StataCorp. College Station, TX: StataCorp LP). Continuous variables were presented as medians with interquartile ranges. Group comparisons were performed using the Chi-Square test for categorical variables or the Mann-Whitney *U* test for continuous variables after testing for normality. Cox regression analysis was used to identify patient and graft survival variables. Variables considered significant in the univariate analysis (*p* < 0.1) were included in the multivariate analysis. Kaplan–Meier survival curves were constructed for factors with statistical significance in the multivariate analysis, defined as a *p*-value < 0.05.

The Hospital das Clínicas da Universidade de São Paulo Ethics Committee approved the project. The retrospective evaluation of patient data in this analysis accords with the declaration of Helsinki.

## Results

**Fig. 1:**
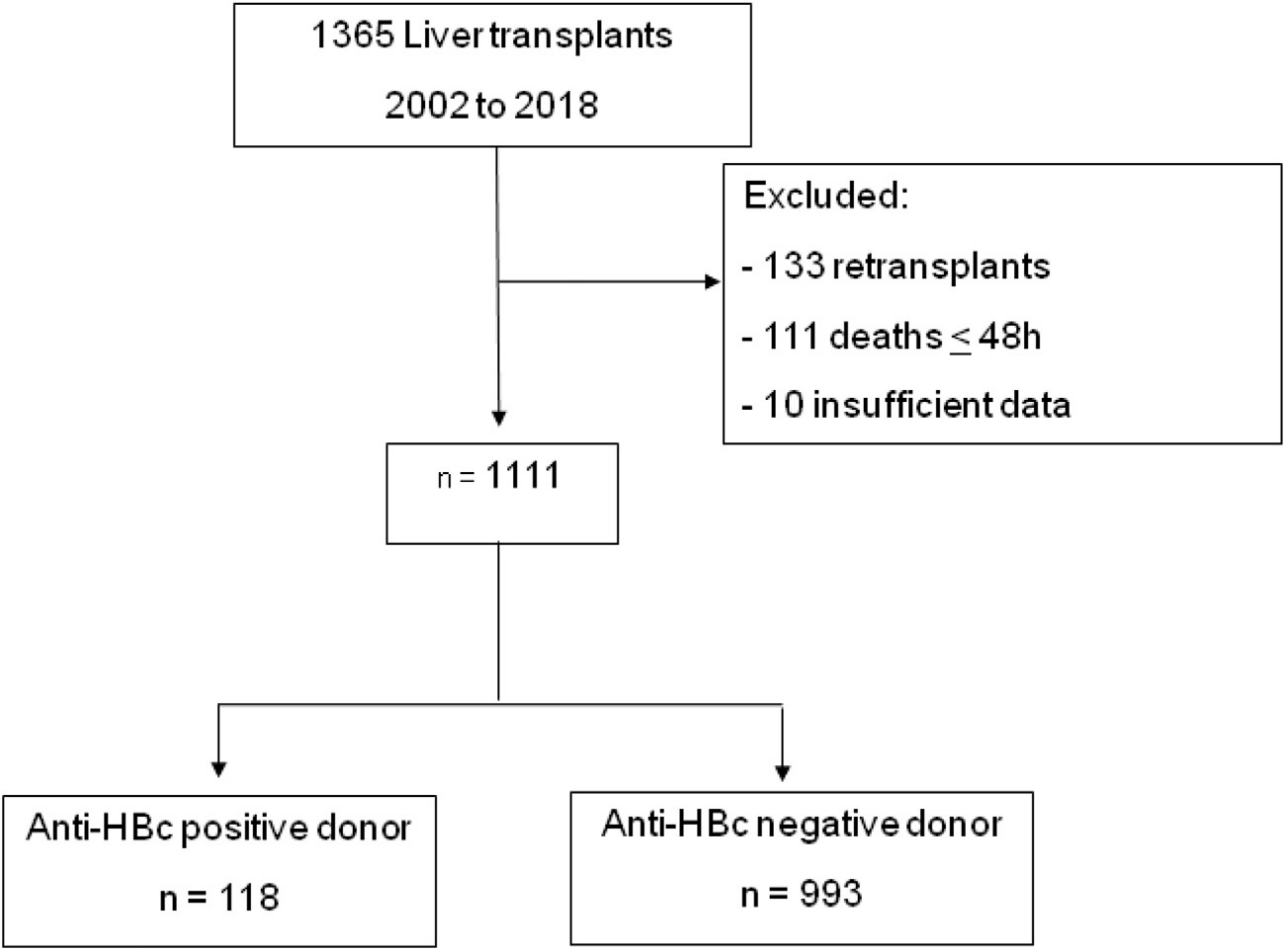
Flow diagram of enrolled patients.

From 2002 to 2018, 1365 liver transplants were performed by the Division of Liver and Digestive Transplants at Hospital das Clínicas, Universidade de São Paulo. Following the inclusion and exclusion criteria as previously described, 1111 patients were selected for analysis (Fig. 1).

### Clinical and demographical characteristics of recipients and donors

Of the 1111 patients, 11 % (*n* = 118) were from anti-HBc positive donors, and 89 % (*n* = 993) were from anti-HBc negative donors. No patient who underwent retransplantation had more than one anti-HBc positive donor. The median follow-up time for all patients was 58 months (IQR 9.7–58.7). Table 1 displays patients' baseline clinical and demographic data in both groups.

**Table 1:** Clinical and demographic data of liver transplant patients according to type of donor (anti-HBc positive or negative), 2002–2018.

	Recipient from anti-HBc positive donor	Recipient from anti-HBc negative donor	*p*-value
	*n* = 118 (11 %)	*n* = 993 (89 %)	
Median age (IQR)	56 (47‒62)	53 (40‒60)	0.001
Male gender	93(79 %)	600 (60 %)	**<0.001**
BMI, median (IQR)	25 (23‒28)	25 (23–29)	0.240
Functional MELD, median (IQR)	15 (11‒21)	20 (13‒31)	**<0.001**
Diabetes mellitus	31/69 (45 %)	188/497 (38 %)	0.256
Etiology			
Chronic Hepatitis C	53 (45 %)	331 (33 %)	0.012
Chronic Hepatitis B	34 (29 %)	69 (7 %)	**<0.001**
Alcoholic Liver Disease	26 (22 %)	205 (20 %)	0.725
Cryptogenic cirrhosis	10 (9 %)	93 (9 %)	0.752
NASH	7 (6 %)	39 (4 %)	0.301
Autoimmune hepatitis	1 (0,8 %)	54 (5 %)	0.030
Primary biliary cirrhosis	1 (0,8 %)	22 (2 %)	0.324
Secondary biliary cirrhosis	1 (0,8 %)	13 (1,3 %)	0.671
Budd Chiari Syndrome	1 (0,8 %)	15 (1 %)	0.568
Biliary atresia	0 (0 %)	4 (0,4 %)	0.490
Caroli Disease	0 (0 %)	6 (0,6 %)	0.397
Wilson's Disease	0 (0 %)	6 (0,6 %)	0.397
Alpha-1 Antitrypsin deficiency	0 (0 %)	2 (0,2 %)	0.626
Hemochromatosis	0 (0 %)	8 (0,8 %)	0.328
Sclerosing Cholangitis	0 (0 %)	21 (2 %)	0.111
Other diagnoses	27 (23 %)	315 (32 %)	0.049
Hepatocellular carcinoma	57/116 (49 %)	323/946 (34 %)	**0.001**
Transplantation year			
2002‒2009	38 (32 %)	305 (31 %)	0.741
2010‒2018	80 (68 %)	688 (69 %)	
Portal vein thrombosis	16/104 (15 %)	96 /657 (15 %)	0.836
Isolated Anti-HBc positive	14 (15 %)	38 (22 %)	0.209
Anti-HBc positive/Anti-HBs > 10	41 (49 %)	67 (40 %)	0.189
Anti-HBc negative/Anti-Hbs > 10	18 (31 %)	200 (75 %)	**<0.001**
Pre-transplante Anti-HBs > 10 UI/mL	67/117 (57 %)	269/702 (38 %)	**<0.001**
Anti-Hbs < 10 UI/mL	51 (43 %)	434 (43 %)	
Anti-Hbs 10‒100 UI/mL	28 (24 %)	110 (11 %)	
Anti-Hbs 100‒1000 UI/mL	26 (22 %)	96 (10 %)	
Anti-Hbs > 1000 UI/mL	13 (11 %)	64 (6 %)	
Retransplantation	3 (2 %)	101 (10 %)	**0.007**
Early Allograft Dysfunction	53 (45 %)	475 (48 %)	0.490
Primary Graft Dysfunction	11/66 (17 %)	64/497 (13 %)	0.395

BMI, body mass index; IQR, interquartile range; MELD, model for end-stage liver disease; NASH, nonalcoholic steatohepatitis.

Values in bold highlight statistically significant results (*p* 0.05).

The analysis of the recipient groups from anti-HBc positive and anti-HBc negative donors showed that the anti-HBc positive group had a significantly higher median age, a higher prevalence of male gender, and a lower functional MELD score. Additionally, the anti-HBc positive recipient group had a higher incidence of hepatocellular carcinoma and positive pre-transplant anti-HBs serology (> 10 IU/mL). The anti-HBc negative donor group had a significantly higher rate of retransplants. Both groups' most prevalent underlying diagnosis was chronic hepatitis C, followed by hepatitis B and alcoholic liver disease. The prevalence of chronic hepatitis B was significantly higher in the anti-HBc positive recipient group (29% vs. 7 %; *p* < 0.001). The majority of transplants occurred between 2009 and 2018 in both groups. The data on early allograft dysfunction and primary graft dysfunction were similar between the groups.

Table 2 presents the donor and intraoperative/perisurgical data. Significantly, the median age of anti-HBc negative donors was lower (38 vs. 50 years, *p* < 0.0001), as well as their BMI and the length of stay in the ICU up to the time of transplantation. DRI was also significantly lower in the anti-HBc negative donor group. The data on donor type (living, deceased, or with familial amyloidotic polyneuropathy) and cold ischemia time were similar between the donor groups.

**Table 2:** Clinical and demographic data of donors according to anti-HBc serology and intraoperative and perioperative data of liver transplants, 2002–2018.

	Anti-HBc positive donor	Anti-HBc negative donor	*p*-value
	*n* = 118 (11 %)	*n* = 993 (89 %)	
Median age (IQR)	50 (42‒56)	38 (24‒51)	**<0.001**
Male gender	69 (58 %)	55 (57 %)	0.725
BMI, median (IQR)	25.5 (23‒27)	25.5 (23‒27.5)	**0.003**
Donor			0.248
Deceased	113 (96 %)	906 (91 %)	
Alive	5 (4 %)	78 (8 %)	
Carrier of FAP	0 (0 %)	6 (1 %)	
Days in ICU (donor)	4 (2–5)	4 (2–5)	0.034
Donor Risk Index	1.58 (1.35‒1.81)	1.42 (1.19–1.69)	**<0.001**
Cold Ischemia Time (hours)	7 (6–8)	7 (5–8)	0.779

BMI, Body Mass Index; FAP, Familial Amyloidotic Polyneuropathy; ICU, Intensive Care Unit; IQR, Interquartile Range.

Values in bold highlight statistically significant results (*p* 0.05).

### Risk factors for post-transplant mortality

Table 3 shows the univariate analysis of risk factors for post-transplant mortality. The median age at the time of transplantation for the patients who died was 52 years (IQR 41‒60), and for the surviving was 54 years (IQR 40‒61; *p* = 0.106). However, when stratifying by age (under 40 years, 40–60 years, and over 60 years), mortality was higher in the groups over 40 years (*p* = 0.086). Significantly, a MELD score of 24 or higher, the need for retransplantation, the occurrence of primary graft dysfunction, and EAD according to Olthoff's criteria, female donor gender, and the length of the donor's ICU stay all correlated with higher mortality in the univariate analysis. Liver transplantation from an anti-HBc positive donor was not associated with an increased risk of death; however, given the relevance of this variable in the present study, it was included in the multivariate analysis, along with all other variables with statistical significance. We performed a survival analysis for the periods before and after 2016 to assess whether there was a difference in survival due to a change in the protocol for transplant recipients of anti-HBc positive donors (inclusion of recipients with anti-HBs positive regardless of anti-HBc status). However, no significant difference was observed (data not shown).

**Table 3:** Univariate analysis of risk factors for post-transplant mortality in patients undergoing liver transplantation, 2002–2018.

	Death		Hazard Ratio (HR) (95 % CI)	*p*-value
	Yes	No		
	(*n* = 398)	(*n* = 713)		
Male gender	261 (66 %)	432 (61 %)	1.16 (0.94–1.43)	0.147
Age			1.13 (0.98–1.30)	**0.086**
<40 years	99 (25 %)	179 (25 %)		
40‒60 years	175 (44 %)	357 (50 %)		
>60 years	124 (31 %)	177 (25 %)		
Functional MELD				
<24	227 (57 %)	481 (68 %)	1	
≥24	170 (42 %)	230 (32 %)	1.49 (1.22–1.81)	**<0.001**
BMI (kg m^-1^²)				
<30	318 (82 %)	582 (81 %)	1	
≥30	70 (18 %)	135 (19 %)	0.80 (0.49–1.31)	0.389
Fulminant hepatitis	36 (9 %)	53 (7 %)	1.19 (0.84–1.68)	0.311
Chronic Hepatitis C	145 (36 %)	239 (33 %)	1.05 (0.85–1.29)	0.620
Chronic Hepatitis B	36 (9 %)	67 (9 %)	0.89 (0.63–1.26)	0.539
Alcoholic Liver Disease	83(21 %)	148 (21 %)	1.00 (0.78–1.27)	0.976
Hepatocellular carcinoma	135 (34 %)	245 (34 %)	0.98 (0.79–1.21)	0.862
Diabetes mellitus	69 (38 %)	150 (39 %)	0.96 (0.71–1.30)	0.814
Portal vein thrombosis	40 (16 %)	72 (14 %)	1.20 (0.85–1.68)	0.292
Transplantation year				
2002‒2009	147 (37 %)	196 (27 %)	1	
2010‒2018	251 (63 %)	517 (73 %)	1.07 (0.87–1.32)	0.496
Cold ischemia time (hours)	7 (5–9)	7 (6–8)	‒	0.281
Retransplantation	60 (15 %)	44 (6 %)	2.20 (1.66–2.90)	**<0.001**
Pre-transplante Anti-Hbs >10UI/mL	112 (40 %)	224 (41 %)	1.00 (0.78–1.27)	0.995
Primary Graft Dysfunction	33 (18 %)	42 (11 %)	1.58 (1.09–2.31)	**0.016**
Early Graft Dysfunction	234 (59 %)	294 (41 %)	1.82 (1.49–2.23)	**<0.001**
Female gender (donor)	185 (47 %)	288 (41 %)	1.23 (1.01–1.50)	**0.038**
Anti-HBc positive Donor	38 (10 %)	80 (11 %)	1.20 (0.86–1.67)	0.281
Donor risk índex	1.46 (1.21–1.70)	1.45 (1.21–1.70)	–	0.275
Days in ICU (donor)	4 (2–5)	4 (3–7)		**0.035**
Donor age				
<50 years	270 (68 %)	500 (71 %)	1	
≥50 years	125 (32 %)	204 (29 %)	1.07 (0.86–1.32)	0.522

BMI, body mass index; ICU, intensive care unit; MELD, model for end-stage liver disease.

Values in bold highlight statistically significant results (*p* 0.05).

**Fig. 2:**
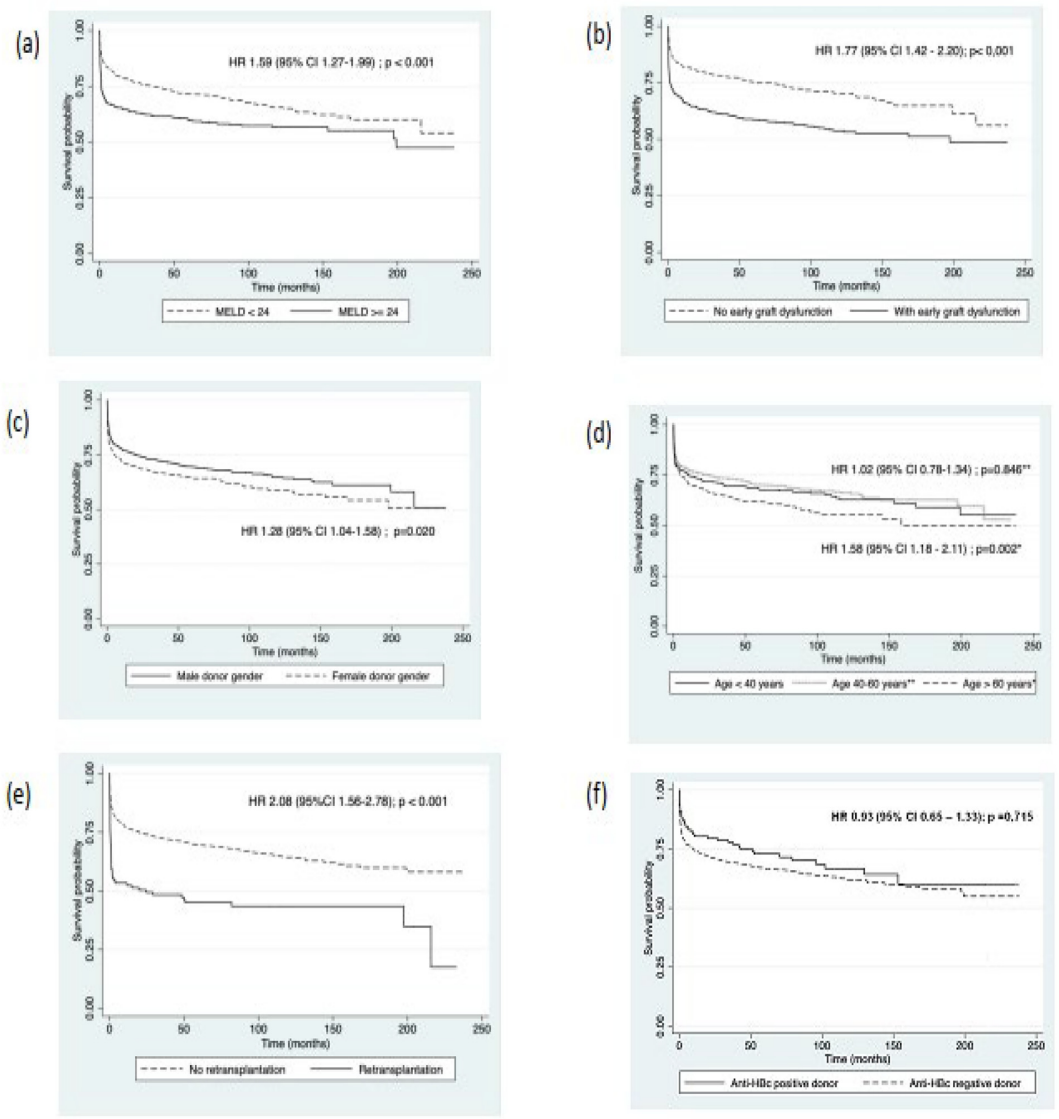
Survival curves after Liver Transplantation according to: (a) MELD score, (b) Early graft dysfunction, (c) Donor gender, (d) Receptor age, (e) Retransplantation, (f) Donor anti-HBc serology, 2002–2018.

Table 4 presents the multivariate analysis of risk factors for post-liver transplant mortality. In the analysis, it was chosen not to include the variable of primary graft dysfunction as it is collinear with early graft dysfunction. Therefore, retransplantation, early graft dysfunction, MELD score of 24 or higher, recipient age over 60 years and female donor gender were associated with a higher risk of death. The number of days the donor spent in the ICU before transplantation and liver transplantation from an anti-HBc positive donor were not risk factors for death in the multivariate analysis. Fig. 2 shows the survival curves of risk factors associated with post-transplant mortality in multivariate analysis.

**Table 4:** Multivariate analysis of risk factors for post-transplant mortality in patients undergoing liver transplantation, 2002–2018.

	Hazard Ratio	95 % CI	*p*-value
Retransplantation	2.08	(1.56–2.78)	**<0.001**
Early graft dysfunction	1.77	(1.42–2.20)	**<0.001**
MELD ≥ 24	1.59	(1.27–1.99)	**<0.001**
Receptor age			
40–60 years	1.02	(0.78–1.34)	0.846
>60 years	1.58	(1.18–2.11)	**0.002**
Donor gender (female)	1.28	(1.04–1.58)	**0.020**
Days in ICU (donor)	0.98	(0.96–1.01)	0.342
Donor anti-HBc positive	0.93	(0.65–1.33)	0.715

ICU, intensive care unit; MELD, model for end-stage liver disease.

Values in bold highlight statistically significant results (*p* 0.05).

### De novo hepatitis b and hepatitis b reactivation

Ten patients experienced *de novo* hepatitis B or HBV reactivation, with 3 cases of *de novo* hepatitis B and 7 cases of reactivation (in chronic hepatitis B carriers). Demographic data, underlying diagnosis, serologies, antiviral and HBIg use, and outcomes are summarized in Table 5. The majority of HBV reactivation and *de novo* hepatitis B occurred in anti-HBc positive recipients.

**Table 5:** Description of cases of HBV reactivation and *de novo* hepatitis B.

N°	Age/ Gender	Disease	Anti-HBc (donor)	Anti-HBc (receptor)	HBIg	Antiviral	Time post-transplant (months)	Reactivation/*de novo* HBV	Death
1	47/M	HBV	–	+	Yes	LAM	15	Reactivation	No
2	58/M	HBV	+	+	Yes	LAM	19	Reactivation	No
3	67/M	HBV	–	+	Yes	LAM	8	Reactivation	Yes
4	28/M	HBV	+	+	Yes	LAM	18	Reactivation	Yes^a^
5	45/F	HBV/HCC	–	+	Yes	LAM	10	Reactivation	Yes^b^
6	69/M	HBV/HCC	–	+	Yes	LAM	17	Reactivation	Yes^c^
7	66/M	HCV/HCC	+	+	No	No	94	*de novo* HBV	No
8	63/M	NASH/HCC	+	+	No	No	78	*de novo* HBV	No
9	39/M	HCV	+	–	No	No	22	*de novo* HBV	No
10	64/M	HBV/HCC	–	+	Yes	ETV	37	Reactivation	No

ETV, entecavir; HBV, Hepatite B; HCV, Hepatite C; HBIg, Hepatitis B Immunoglobulin; HCC, hepatocellular carcinoma; LAM, lamivudina; NASH, nonalcoholic steatohepatitis*.*

a Transplant in 2007; deceased in 2020 due to COVID-19.

b Transplant in 2008; deceased in 2009 due to hepatocellular carcinoma.

c Transplant in 2011; deceased in 2013 due to hepatocellular carcinoma.

**Fig. 3:**
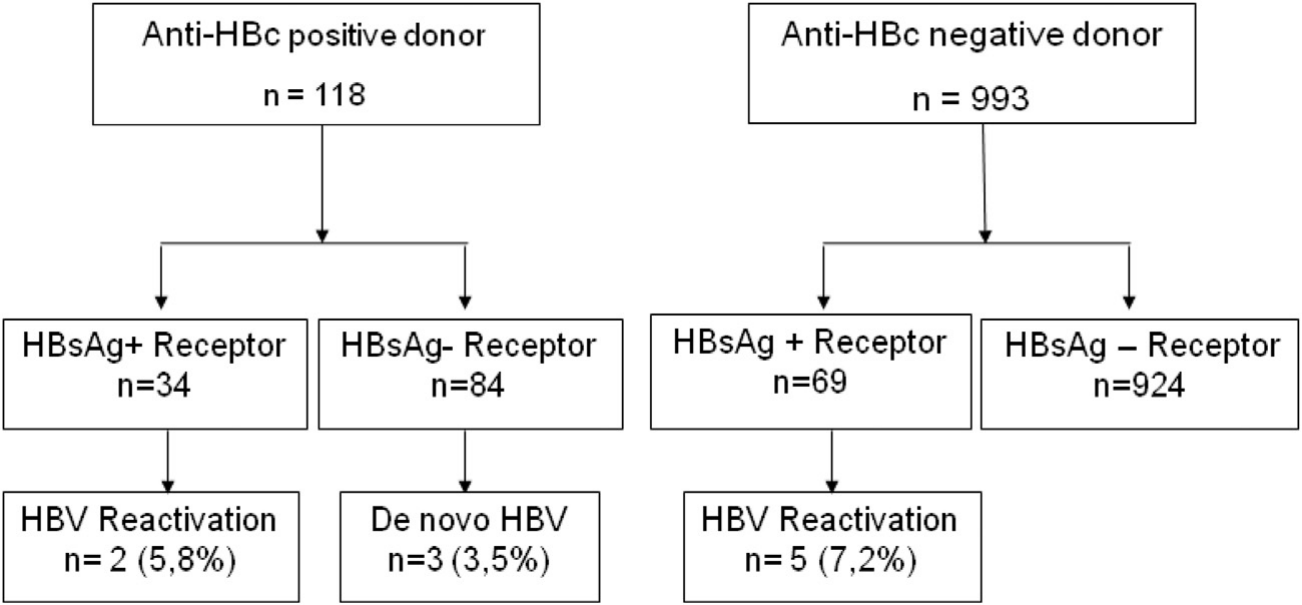
Risk of reactivation and *de novo* hepatitis B in liver transplant recipients according to donor-anti-HBc serology and receptor-HBsAg serology.

The risk of reactivation or *de novo* hepatitis B in liver transplant recipients from anti-HBc-positive donors with chronic hepatitis B and HBsAg-negative recipients was 5.8 % and 3.5 %, respectively (Fig. 3). Three recipients without prior hepatitis B who developed de novo hepatitis B did not receive any antiviral prophylaxis, and 2/3 had reactive anti-HBs before transplantation. Among recipients from anti-HBc-negative donors, the risk of hepatitis B reactivation among chronic carriers was 7.2 %.

No recipient underwent retransplantation, and half of them had EAD. All recipients with chronic hepatitis B received HBIg appropriately, following the current institutional protocol. Three patients did not receive prophylactic antiviral treatment until the occurrence of *de novo* hepatitis B (2 cases of hepatitis C and one with NASH). All patients who received antivirals and experienced reactivation or *de novo* hepatitis B were under lamivudine, except one who irregularly used entecavir. No patient experienced reactivation while on tenofovir. Four patients died, one due to COVID-19 complications and two due to hepatocellular carcinoma recurrence. Of note, one of these patients already had tumor vascular invasion in the explant. One patient died, and the cause could not be determined (patient 3).

## Discussion

The organ demand for transplantation exceeds the supply, resulting in prolonged waiting times for patients on the transplant list worldwide, and there is increasing use of graft from antiHBc-positive donors. Our study compared the survival of patients who underwent liver transplants using donors with positive anti-HBc serology to those with negative anti-HBc serology over a 16-year period. The similarity in survival between the two groups suggests that using liver grafts from anti-HBc positive donors is a safe option to increase the pool of potential donors without increasing the mortality rate.

Literature data on the survival of liver transplant recipients from anti-HBc positive donors are controversial in studies conducted in the 1990s before the routine adoption of prophylaxis with antivirals and HBIg. However, it is important to note that even in these studies, graft loss was not related to HBV reactivation or *de novo* HBV occurrence but rather to postoperative clinical and surgical complications,^3^ suggesting that the presence of anti-HBc may be a marker of suboptimal graft quality and greater severity in recipients.^4^^,^^10^, ^11^, ^12^

Wong and colleagues^5^ also evaluated the survival of liver transplant recipients from anti-HBc positive donors in a high-prevalence HBV area. They found no difference in survival compared to recipients from anti-HBc negative donors. In this cohort, factors associated with lower recipient survival were recipient male gender, transplantation for hepatocellular carcinoma, prolonged cold ischemia time, and surgical complications. Other studies and meta-analyses have also found similar data regarding the safety of using anti-HBc positive grafts with adequate antiviral prophylaxis.^2^^,^^6^^,^^7^^,^^12^^,^^13^

Our study identified classical factors related to lower survival, as previously described in other studies, such as retransplantation, high MELD score, recipient age over 60 years, and early allograft dysfunction.^14^, ^15^, ^16^, ^17^

Furthermore, we also found that the female gender of the donor was also related to lower survival. Several studies have linked lower graft and patient survival in male recipients of female donor livers, especially in younger female donors.^18^, ^19^, ^20^ In Lee and colleagues' study, the risk of retransplantation or death was significantly higher in male recipients from female donors, but no difference in survival was found when the donor was 40 years or older.^20^ One hypothesis for this lower graft survival from female donors suggests the involvement of the lack of estrogen and progesterone hormones in male liver recipient patients, combined with numerical differences in hepatic estrogen and androgen receptor expression between men and women and an increase in enzymes related to microsomal oxidative stress in men.^18^

Early allograft dysfunction, according to Olthoff criteria,^9^ was associated with lower survival in our case series, and its occurrence was considered high in both the group of patients who died and those who did not (59 % and 41 %, respectively). The quality of the donor's liver is directly related to the success of the transplant and long-term survival. In general, the occurrence of EAD depends on factors related to donor care (BMI, steatosis, cold ischemia time), recipient factors (MELD score, hepatocellular carcinoma), and surgical time (prolonged surgical time, intraoperative blood transfusion).^21^ In the validation study by Olthoff and colleagues,^9^ the overall occurrence of EAD was 23.2 %, and associated risk factors included donor age, MELD score, recipient age, and donor BMI. Zhu et al. found an overall prevalence of 45.3 % for EAD; 6-month survival was significantly lower in the group that experienced dysfunction compared to the group that did not experience early graft dysfunction (77.8% vs. 98.9 %, respectively – *p* < 0.001).^21^ Multivariate analysis in this series showed, among other factors, that female donor gender was related to a higher occurrence of EAD. Hoyer et al. found a 38.7 % occurrence of EAD in a transplant center in Germany, and factors associated with a higher occurrence included donor BMI, elevated gamma-glutamyl transferase levels, macrovesicular steatosis, and cold ischemia time.^22^

Our study identified seven cases of HBV reactivation in chronic carriers and three cases of *de novo* hepatitis B (Fig. 2). The risk of reactivation or *de novo* hepatitis B in liver transplant recipients from anti-HBc-positive donors was considered low and varied depending on the recipient's prior serological status, with a higher risk observed in patients with chronic hepatitis B compared to HBsAg-negative recipients (5.8 % and 3.5 %, respectively). In the literature, the risk of post-transplant *de novo* hepatitis B varies depending on the studied population and the prophylaxis used,^6^ with incidences ranging from 0 % to 50 %.^23^, ^24^, ^25^ The prophylaxis used in our centers consisted of HBIg for one year in chronic HBV carriers, combined with antiviral medication or monoprophylaxis with antiviral agents (currently tenofovir or entecavir) for HBsAg-negative patients. Except for one patient who experienced HBV reactivation while irregularly using entecavir, all other patients who developed HBV post-transplant used either lamivudine (6 cases) or no antiviral medication (3 cases). In our hospital, lamivudine was discontinued for both prevention and treatment of hepatitis B due to its low genetic barrier and frequent occurrence of resistance over time.

Jung et al., in a 30-year follow-up cohort, found 13.8 % of *de novo* hepatitis B in a population of 152 HBsAg-negative liver transplant recipients from anti-HBc-positive donors. In this study, prophylaxis was performed with HBIg as monotherapy, and no patient died or experienced graft loss due to hepatitis B.^26^ Another study in a high-prevalence area found *de novo* HBV in only 3/108 (2.8 %) patients who received lamivudine prophylaxis.^5^ A meta-analysis published by Cholongitas et al.^2^ found HBV reactivation in 11 % of HBsAg-positive recipients. *De novo* HBV occurred in 19 % of HBsAg-negative recipients, being more common in recipients who were anti-HBc negative/anti-HBs negative (without prophylaxis) than in recipients who were anti-HBc positive and anti-HBs reactive (48% vs. 15 %, *p* < 0.001). Despite the presence of anti-HBs reducing the rate of *de novo* HBV, it is not eliminated in the absence of appropriate antiviral prophylaxis.

This study, to the best of our knowledge, is the largest Brazilian single-center cohort study that evaluates the survival and risk of hepatitis B reactivation and *de novo* hepatitis B in liver transplant recipients from anti-HBc-positive donors. The data encourage various transplant services in the country to use anti-HBs-positive grafts safely. Additionally, the study provides important information regarding risk factors for post-transplant mortality, including the not frequently assessed EAD factor.

Limitations of the study include its retrospective and single-center nature, the lack of data on immunosuppressant use and rejection, which could impact both survival and the occurrence of hepatitis B reactivation.

In conclusion, our study showed that using liver grafts from donors with positive anti-HBc serology is safe, especially when using second-generation antivirals (tenofovir and entecavir) in combination with HBIg (when indicated). Furthermore, the occurrence of reactivation or *de novo* hepatitis B was considered low and easily manageable with antiviral therapy. These findings contribute to expand donor options and improve patient outcomes in liver transplantation.

## Funding

This research did not receive any specific grant from funding agencies in the public, commercial, or not-for-profit sectors.

## Conflicts of interest

The authors declare that the research was conducted in the absence of any commercial or financial relationships that could be construed as a potential conflict of interest.
